# Profound loss of neprilysin accompanied by decreased levels of neuropeptides and increased CRP in ulcerative colitis

**DOI:** 10.1371/journal.pone.0189526

**Published:** 2017-12-12

**Authors:** Zeynep Gök Sargın, Nuray Erin, Gokhan Tazegul, Gülsüm Özlem Elpek, Bülent Yıldırım

**Affiliations:** 1 Department of Internal Medicine, Division of Gastroenterology, Akdeniz University Faculty of Medicine, Antalya, Turkey; 2 Department of Medical Pharmacology, Akdeniz University Faculty of Medicine, Antalya, Turkey; 3 Department of Pathology, Akdeniz University Faculty of Medicine, Antalya, Turkey; "INSERM", FRANCE

## Abstract

Neprilysin (NEP, CD10) acts to limit excessive inflammation partly by hydrolyzing neuropeptides. Although deletion of NEP exacerbates intestinal inflammation in animal models, its role in ulcerative colitis (UC) is not well explored. Herein, we aimed to demonstrate changes in NEP and associated neuropeptides at the same time in colonic tissue. 72 patients with UC and 27 control patients were included. Patients’ demographic data and laboratory findings, five biopsy samples from active colitis sites and five samples from uninvolved mucosa were collected. Substance P (SP), calcitonin gene related peptide (CGRP) and vasoactive intestinal peptide (VIP) were extracted from freshly frozen tissues and measured using ELISA. Levels of NEP expression were determined using immunohistochemistry and immunoreactivity scores were calculated. GEBOES grading system was also used. We demonstrated a profound loss (69.4%) of NEP expression in UC, whereas all healthy controls had NEP expression. Patients with UC had lower neuronal SP; however non-neuronal SP remained similar. UC patients had also lower neuronal and non-neuronal VIP levels. CGRP were low in general and no significant changes were observed. Additionally, CRP positive patients with UC had higher rates of NEP loss (80% vs 51.9%) and lower SP levels when compared with CRP negative patients with UC. Concurrent decreases in SP and VIP with profound loss of NEP expression observed in UC is likely to be one of the factors in pathogenesis. Further studies are required to define the role of neuropeptides and NEP in UC.

## Introduction

Ulcerative colitis (UC) is characterized by chronic idiopathic inflammation of the colonic mucosa. The surface enzyme neprilysin (also known as neutral endopeptidase or CD10, NEP) hydrolyzes neuropeptides including substance P (SP), calcitonin gene related peptide (CGRP) and vasoactive intestinal peptide (VIP) [1]. Deletion of NEP as well as treatment with a NEP inhibitor exacerbates intestinal inflammation induced by *C*.*difficile toxin-A* [2]. Furthermore, inflammation was prevented in NEP knock-out mice by administration of recombinant NEP [2]. Although NEP may act as an important anti-inflammatory molecule, its role in inflammatory bowel diseases such as in UC is not well explored. Loss of NEP immunoreactivity in a limited number of patients (n = 18) with this disease was demonstrated in a recent study by Lloyd and Owens [3].

Neuropeptides hydrolyzed by NEP were previously shown to be involved in gastrointestinal motility, secretions and inflammation [4]. Of these peptides, SP is a tachykinin expressed collectively by the enteric nervous system, enteroendocrine cells and immune mucosal cells. SP receptors, neurokinin receptors -1, -2 and -3, are found on vasculature, muscular, epithelial and immune cells throughout the gastrointestinal system [4]. Although there has been a great interest on a possible role of SP and its receptors in the pathogenesis of UC, conclusive results are lacking. Specifically, a majority of studies show increased SP expression in UC patients which correlates with disease activity [5–9]. In contrast, several studies demonstrated a reduction in SP levels in UC [10,11]. Findings also suggest that SP-induced activation of the neurokinin receptor-1 (NK1R) may also have a protective role in the recovery phase of UC [12]. According to our knowledge, there are no previously published studies examining changes in levels of SP and NEP [4].

VIP, another substrate for NEP, is abundantly expressed in the myenteric plexus, and also, in T cells and eosinophils, revealing a potential role in the inflammatory response [12,13]. VIP predominantly acts as an anti-inflammatory molecule affecting both innate and adaptive immunity. There are few studies examining changes in VIP levels in UC [14–18] which showed consistent decrease suggestive of a possible therapeutic value of a VIP mimetic. In each study reported, approximately 20 to 60 patients were examined. Herein, changes in VIP levels were examined in relation to changes in NEP and other neuropeptides in a larger population of 72 UC patients.

CGRP is another neuropeptide hydrolyzed by NEP. Studies on CGRP levels on UC are scarce and inconsistent [10,18]. Hence we here also examined changes in CGRP levels in our patient population.

The reason for inconsistent findings regarding mucosal neuropeptides levels in UC may result from differences in patient populations and limitations of the methods used. Herein, we aimed to identify simultaneous changes in NEP and associated neuropeptides in colonic tissue using a quantitative approach developed by Erin et al [19] which can, in part, discriminate peptides localized in sensory nerve fibers and in non-neuronal tissue. This method was chosen because neuronal fractions of neuropeptides may have distinct functions that differ from neuropeptides found in non-neuronal tissues [17]. This method was not used previously to demonstrate changes in neuropeptide levels in UC.

## Patients and methods

This study was approved by the Akdeniz University Clinical Studies Scientific Ethics Committee (Date approved: 01.03.2011. Approval number: 32. Document number: B.30.2.AKD.0.20.05.05/32). After ethical approval, seventy two patients with UC and twenty seven control patients between ages of 18 and 75 were enrolled in this study. All patients were informed of the study protocol verbally and in writing regarding the aim of the study, possible outcomes and adverse events that may occur during, and after, the procedure, with a signed informed consent. Sample size was calculated based on a study conducted by Lloyd and Owens [3], where NEP expression was lost by 20–80% in patients with UC. Sample size was calculated as twenty three patients per group, with an expected 50% loss of NEP expression in patients with UC (90% power and %95 confidence levels). However, since the previously reported range of NEP loss was broad, we included more patients with UC to provide for more conclusive results.

Patients with ulcerative colitis presenting with abdominal pain and rectal bleeding were chosen randomly from patients admitted to the Gastroenterology outpatient clinic. Patients in the control group were negative for gastrointestinal diseases and included those undergoing colonoscopy for other indications such as iron deficiency anemia, malignancy check-up, or possible gastrointestinal bleeding. All patients in both groups provided blood samples for the study. Patients were excluded with known malignancies, chronic liver disease, chronic kidney disease, congestive heart failure, hepatitis B, hepatitis C or human immunodeficiency virus positivity, and those with other known systemic autoimmune conditions, severe sepsis, septic shock and patients with unstable clinical conditions.

Age, gender, hemoglobin (g/dL), white blood cell (WBC) count (x10^9^/L), c-reactive protein (CRP) (mg/dL), alanine aminotransferase (ALT) (U/L), creatinine (mg/dL) and sedimentation rate (mm/hr) were documented at the day of colonoscopy. During colonoscopy, five biopsy samples from active colitis sites and, when possible, five samples from uninvolved mucosa were collected (healthy site). For the control group, only five biopsies from healthy sites were performed. Three biopsy materials were frozen with liquid nitrogen for measurement of neuropeptide levels; other two samples were fixed with formalin for histopathological analysis.

Samples frozen with liquid nitrogen were kept at -80°C for later measurements. Extraction of neuropeptides was performed using acetic acid as described before [20]. Two-step acetic acid extractions were performed to estimate neuronal and non-neuronal components of neuropeptides as described previously by Erin et al [19]. Using this method SP eluding within the first 10 minutes of extraction (first extraction) originates mostly from capsaicin-sensitive sensory nerve endings whereas the second extraction contains predominately non-neuronal SP. Biopsy samples were cut into small pieces and kept in 1 ml of 2% acetic acid at 95°C for 10 minutes then centrifuged and the supernatants assayed for the first extraction. Tissues were reincubated in 1 ml of 2% acetic acid at 95°C for 50 min and supernatants for the second extraction. Supernatants were dried completely in a speed-vacuum and reconstituted in 300 μl of sample buffer of SP EIA kit (Cayman, CatNo: 1800-364-9897). From each sample 25 and 50 μl were used for immunoassay, which mostly gave results within confidence interval of 95%. If necessary, further dilutions were tested. SP, VIP and CGRP levels were measured using commercially available ELISA kits using the same method (SP, Cayman, CatNo: 1800-364-9897; VIP, Bachen-Pennisula Lab LLC CatNo: 823513; CGRP, Phoenix CatNo: 601068).

For immunohistochemisty, staining was conducted via a closed-system immunohistochemistry staining automatizer (Benchmark XT, Ventana, Roche, USA), using a NEP primary antibody (anti-CD10, Clone: 2A1H5E1, Thermo Fisher Scientific Inc, UK). Formalin-fixed paraffin-embedded tissues were deparaffinized and heated for antigen retrieval. Tissues were incubated in 3% hydrogen peroxide in methanol to block endogenous peroxidase. After washing and incubation with primary antibody, tissues were reacted with a secondary biotinylated antibody. After washing, horse radish peroxidase-streptavidin conjugates were added. 3'-Diaminobenzidine and Mayer's hematoxylin were used for color development (All consumables apart from primary antibody were purchased from Ventana, Roche, USA for closed system automatizer). Negative controls were created by omitting the primary antibody from the reaction. Positive controls were created from healthy kidney tissue which express high levels of NEP. Intensity and area of staining was graded and immunoreactivity scores (IRS) calculated as described previously [21]. An immunoreactivity scale with a 4-tier grading system (0—negative, 1—weak, 2—moderate, and 3—strong staining intensity) was used. Besides intensity, area of staining was also graded as follows: Percentage scale: 0—no stained cells; 1—>0% to 25% stained cells; 2—>25% to 50% stained cells; 3—>50% to 75% stained cells; and 4—>75% to 100% stained cells. The immunoreactivity score (IRS) was calculated using IRS = SI×PS; where PS is score for percent of staining and SI is staining intensity. Histopathological GEBOES grading system, which is a reproducible grading scale for histological assessment of inflammation in ulcerative colitis, was also used [22]. An Olympus BX51 microscope was used for analysis (Olympus Life Sciences, Japan). Histopathological and immunoreactivity scores were calculated and reported by an experienced pathologist blinded to the patient records and primary study hypothesis.

SPSS 11.0 was used for statistical analysis. Categorical variables were analyzed and presented as frequency and percentages, chi-square tests were used for statistical significance. Continuous variables were analyzed and presented as either mean and standard error or median and range; Mann–Whitney-U test was used to determine significance with *P* values less than 0.05 considered significant.

## Results

### Demographics

There were 72 colitis and 27 control patients enrolled in the study. Demographics and blood tests included age, gender, new diagnosis of UC or reactivation, hemoglobin, WBC count, CRP, ALT, creatinine and sedimentation rate (Table 1). Subgroup analysis for age was done by creating two groups: 45 years old and lower and older than 45 years old. Patients in UC group were divided into two sub-groups by their duration of disease: patients with newly diagnosed UC were categorized as new patients; patients with longer than 1 year history of UC were categorized as re-activation. Patients with UC were also divided into two categories by colonoscopy findings: distal colitis and pancolitis. CRP positivity was defined as a value higher than 0.5 mg/dL. Sedimentation positivity was defined according to age and gender [positive for male patients: > age/2, for female patients: > (age/2) +10].

**Table 1:** Distribution of clinical and laboratory parameters of patients with ulcerative colitis and healthy controls.

		Healthy (n = 27)	All colitis (n = 72)	Distal colitis (n = 30)	Pancolitis (n = 42)
**Age**		50±15	43±14	46±13	41±14
**Age Groups**	40 and lower	8 (29.6%)	32 (44.4%)	11(36.6%)	21 (50%)
	Higher than 40	19 (70.4%)	40 (55.6%)	19 (63.4%)	21 (50%)
**Gender (M/F)**		11/16	40/32	20/10	22/20
**Past history**	New diagnosis	-	30 (41.7%)	16 (53.3%)	14 (31.8%)
	Reactivation	-	42 (58.3%)	14 (46.7%)	30 (78.2%)^+++^
**Hemoglobin**	g/dL	13.1±1.48	12.1±2.01*	12.9±1.94	11.6±1.91^++^
**WBC count**	x10^9^/L	7.3±1.6	8.9±3.1**	8.3±2.2	9.3±3.6^+^
**ALT**	U/L	16±5	17±9	20±9	15±8
**Creatinine**	mg/dL	0.75±0.15	0.72±0.14	0.72±0.14	0.72±0.15
**CRP**	mg/dL	0.16±0.13	2.3±3.5***	0.81±1.1	3.4±4.1^+++^
**CRP (+)**	(>0.5 mg/dL)	0 (0%)	45 (62.5%)***	13 (43.3%)	32 (76.1%)^+++^
**Sedimentation**		18±15	35±25***	20±14	45±27^+++^
**Sedimentation (+)**	Corrected by age and gender	5 (18.5%)	46 (63.8%)***	12 (40%)	34 (81%)^+++^
**GEBOES grade**		1.48±0.93	4.54±0.76***	4.53±0.89	4.54±0.67

*P<0.05

**P<0.01

***P<0.001

All colitis compared with healthy controls. Chi-square test was used for categorical variables, Mann–Whitney-U test was used for continuous variables.

^+^*P*<0.05

^++^*P*<0.01

^+++^*P*<0.001

Pancolitis compared with distal colitis. Chi-square test was used for categorical variables; Mann–Whitney-U test was used for continuous variables.

Colitis and control groups were similar regarding age groups and gender. Reactivation was more common in patients with pancolitis. Anemia, leukocytosis, higher CRP and sedimentation positivity were seen in colitis. Patients with pancolitis had more pronounced anemia, leukocytosis and higher CRP and sedimentation than distal colitis. GEBOES grade was higher in colitis when compared with controls, as expected. Patients with distal colitis and pancolitis had similar GEBOES grades (Table 1).

### Changes in NEP expression

**Fig 1:**
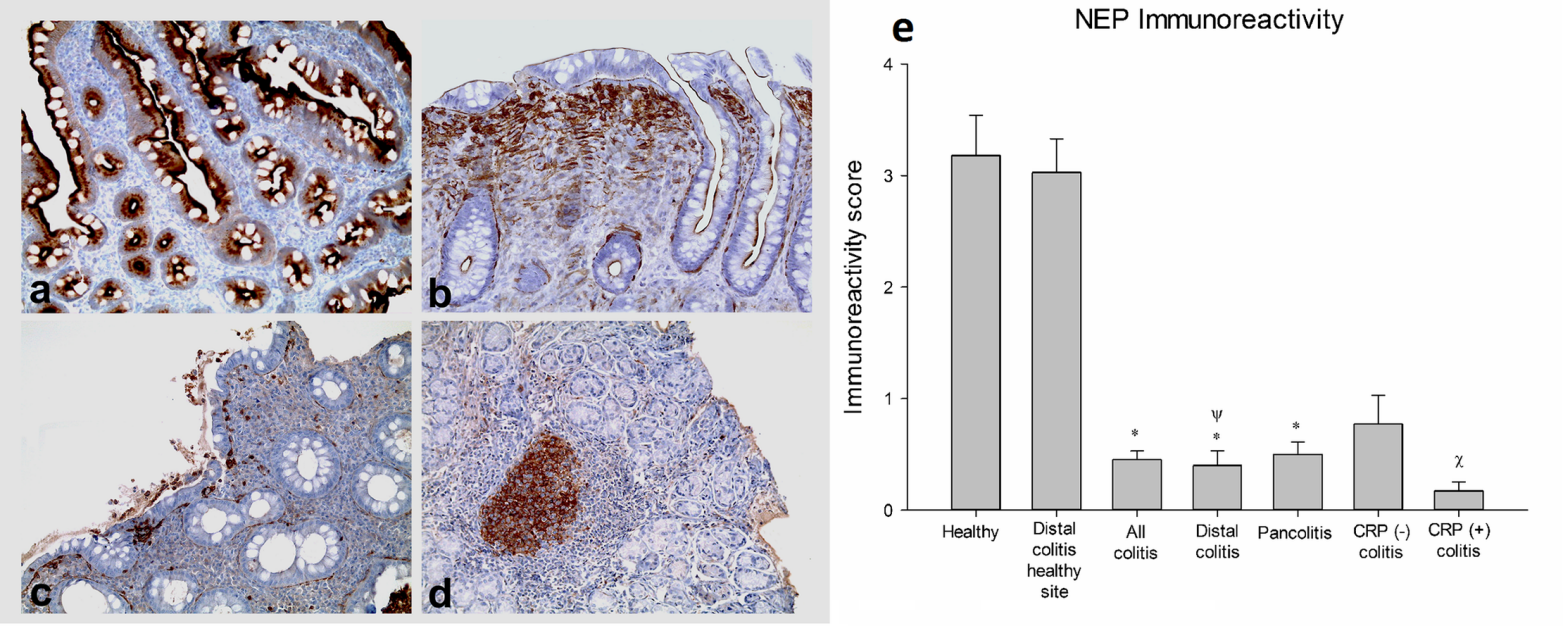
NEP expression in colonic mucosa. **a:** Normal mucosa from a healthy subject. **b:** Uninvolved mucosa from a case of distal colitis. **c:** Mucosa from a case with distal colitis **d:** Inflamed mucosa in an area of colitis from a case with pancolitis. There is considerable negative staining in colitis. (Magnifications: a, b and c x100; d x50; CD10 staining counterstained with haematoxylin) e: Immunoreactivity score of neprilysin (mean ± SEM). *P<0.05, compared to healthy tissue. Ψ *P*<0.05, compared to distal colitis healthy site. Х *P*<0.05 compared to CRP negative patients with UC.

NEP expression was determined using immunohistochemistry. Specifically NEP staining intensity and percent of staining were analyzed and immunoreactivity scores were calculated. A profound loss of NEP expression was determined in patients with colitis (Fig 1A–1D, Table 2). Overall changes in immunoreactivity scores were shown in Fig 1E.

**Table 2:** Distribution of NEP positivity of patients with ulcerative colitis and healthy controls.

NEP Positivity	Healthy (n = 27)	Distal colitis healthy site (n = 30)	All colitis (n = 72)***	Distal colitis (n = 30)	Pancolitis (n = 42) ^+^
**No staining**	0 (0%)	0 (0%)	50 (69.4%)	22 (73.3%)	28 (66.6%)
**1+**	11 (40.7%)	13 (43.3%)	18 (25%)	6 (20%)	12 (28.5%)
**2+**	10 (37%)	11 (36.6%)	4 (5.6%)	2 (6.7%)	2 (4.8%)
**3+**	6 (22.3%)	6 (20%)	0 (0%)	0 (0%)	0 (0%)

***P<0.001, All colitis compared with healthy controls. Chi-square test was used for categorical variables.

^+^*P*<0.05. Pancolitis compared with distal colitis. Chi-square test was used for categorical variables.

We additionally observed that CRP positive UC patients had higher rates of NEP loss [80% in CRP (+) patients with UC vs 51.9% in CRP (–) patients with UC] (p<0.05, Chi-square test) (Fig 1E). Other subgroups such as age, gender and new diagnosis/reactivation had similar levels of NEP expression.

### Changes in SP levels

**Fig 2:**
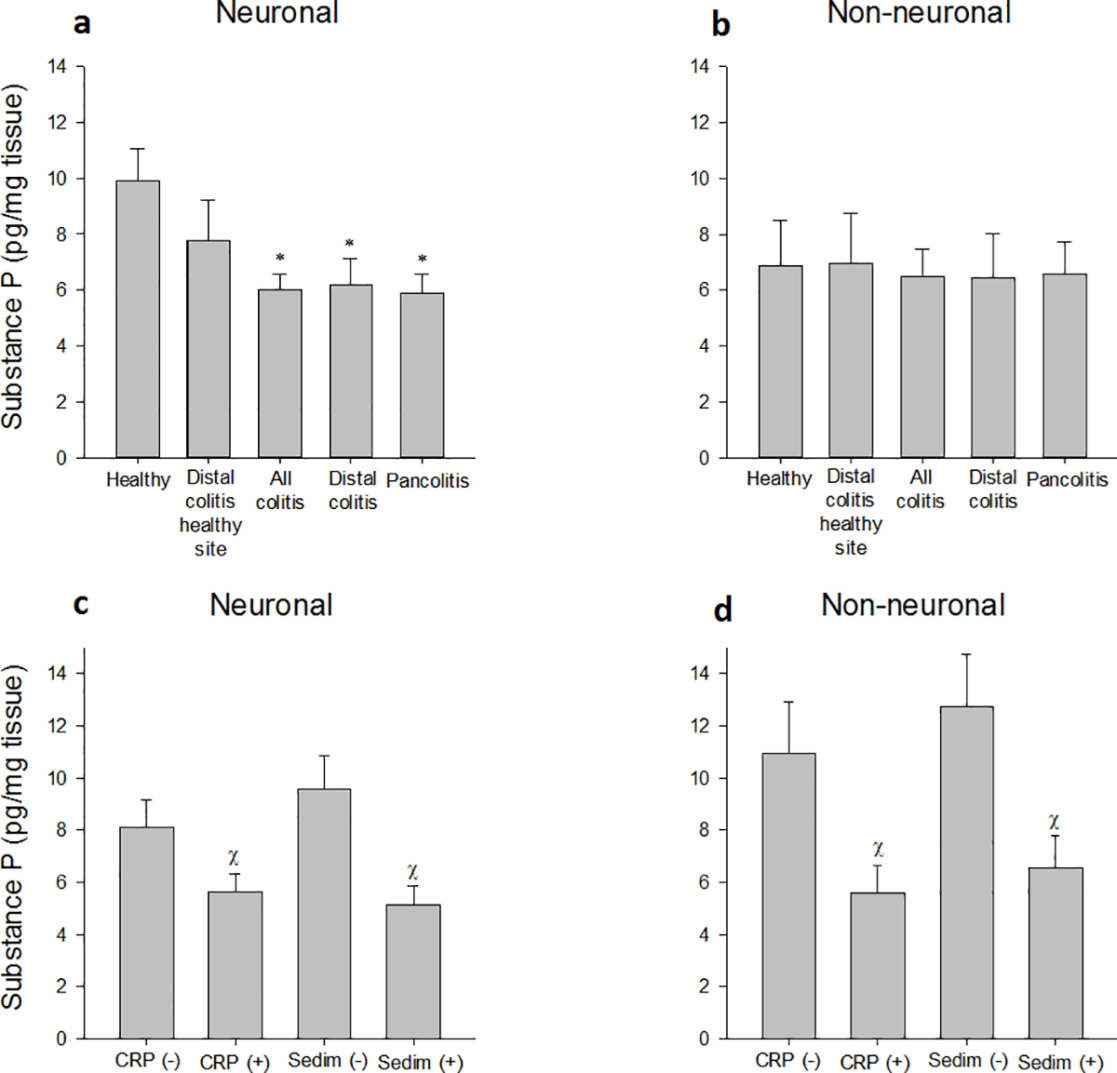
Changes in substance P levels (mean ± SEM). **a:** Neuronal and **b:** Non-neuronal SP levels were demonstrated. *P<0.05, compared to healthy tissue. In patients with UC, **c:** Neuronal and **d:** Non-neuronal SP levels were reduced with CRP and sedimentation positivity. Х *P*<0.05 compared to CRP and sedimentation negative patients, respectively.

Neuronal and non-neuronal SP levels were similar between healthy controls and healthy tissue samples of distal colitis. Patients with distal colitis and pancolitis had lower neuronal SP; however non-neuronal SP levels were similar with the control group (Fig 2A and 2B). In accordance, UC patients with CRP or sedimentation positivity had lower neuronal and non-neuronal SP levels (Fig 2C and 2D). Other subgroups such as age, gender and new diagnosis/reactivation had similar SP levels.

### Changes in VIP levels

**Fig 3:**
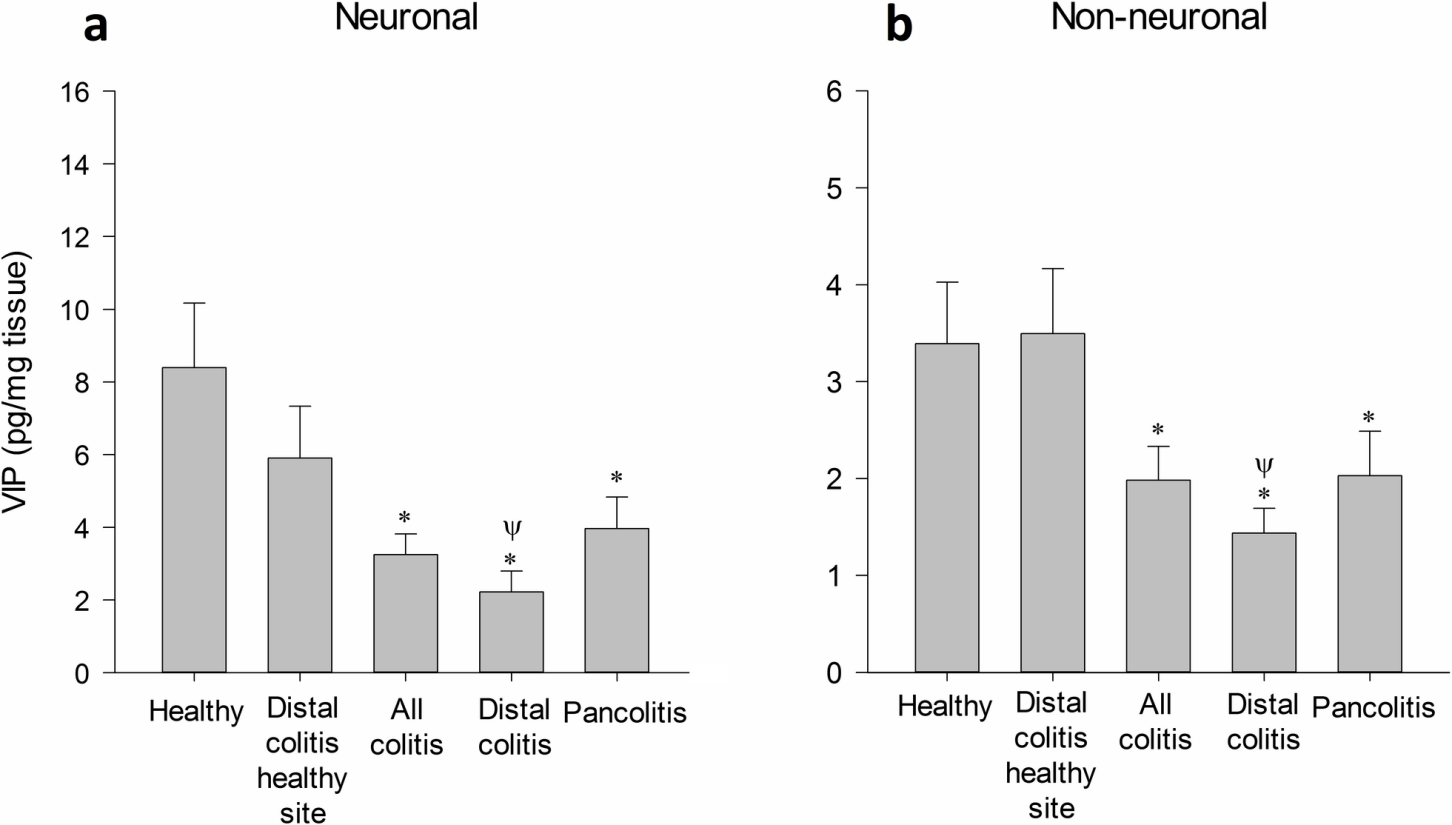
Changes in VIP levels (mean ± SEM). **a:** Neuronal and **b:** Non-neuronal levels of VIP are demonstrated. *P<0.05, compared to healthy tissue. Ψ *P*<0.05, compared to distal colitis healthy site.

Healthy controls and healthy tissue samples of distal colitis had similar levels of neuronal and non-neuronal VIP. Samples from patients with distal colitis and pancolitis had lower neuronal and non-neuronal VIP when compared with the control group. Active inflammatory sites of patients with distal colitis had lower VIP compared to healthy tissue samples of distal colitis (Fig 3A and 3B). Subgroup analyses for age, gender, CRP positivity, sedimentation positivity and new diagnosis/ reactivation were similar.

### Changes in CGRP levels

Neuronal and non-neuronal CGRP were low in general (2±0.5 pg/mg tissue) and were similar between healthy controls, healthy tissue samples of distal colitis and colitis (S1 Fig.)

## Discussion

In this study, changes in the endopeptidase NEP, and neuropeptides hydrolyzed by NEP in UC were evaluated for the first time. A profound loss of NEP in UC was observed, which was more pronounced with CRP positivity. In addition, patients with colitis had lower neuronal SP levels, whereas non-neuronal SP was similar. Neuronal SP levels were even lower in patients with CRP or sedimentation positivity. Both neuronal and non-neuronal VIP levels were reduced in patients with UC.

Deletion of NEP, the enzyme that hydrolyzes neuropeptides, leads to neurogenic inflammation, which is alleviated by administration of a NK1R antagonist [23]. Kirkwood et al [2] demonstrated both NEP knock-out mice and mice treated with NEP inhibitor had exacerbated intestinal inflammation induced by *C*.*difficile toxin A*. Inflammation was prevented in NEP knock-out mice by administration of recombinant NEP. Lloyd and Owens [3] demonstrated varying levels of mucosal NEP loss in 8 patients with UC. We here observed complete loss of NEP expression in 69.4% of 72 patients with UC. Furthermore, CRP positive patients with UC had more pronounced NEP loss when compared with CRP negative UC patients (80% vs 51.9%). Therefore, a profound loss of NEP may be associated with the pro-inflammatory condition of colonic mucosa.

Neuropeptides hydrolyzed by NEP were previously shown to be involved in gastrointestinal motility, secretions and inflammation [4]. Of these neuropeptides, SP is a pro-inflammatory mediator. SP directly and indirectly (via IL-1B, IL-6 and IL-8) causes chemotaxis of neutrophils and modulates activation, promotes survival and enhances phagocytic ability of innate immune cells [12]. The main SP receptor, NK1R is responsible for the pro-inflammatory effects [4]. The extraction model used here partly differentiates levels of SP found in sensory nerve endings as described previously [19]. It is interesting that neuronal SP levels were decreased in UC which actually indicate loss of SP-containing sensory neurons. Although colonic sensory neurons containing SP play a role in neurogenic inflammation [24], these neurons are also involved in eradication of pathogenic bacteria [25] such as *Salmonella* [26]. Because *Salmonella* infection is a clear risk factor for inflammatory bowel disease [27], decreased levels of SP-containing nerve fibers may contribute to the pathogenesis of UC. This idea is further supported with our finding that neuronal SP levels were even lower in patients with CRP or sedimentation positivity. Non-neuronal SP levels were not altered in UC but subgroup analysis yield that UC patients with CRP or sedimentation positivity had lower non-neuronal SP levels which likely to be present in the colonic epithelial cells [19]. This decrease observed here might be due to loss of colonic epithelial cells in severe UC with CRP and sedimentation positivity.

However, majority of studies show increased levels or expression of SP in UC patients which also correlated with disease activity [5–9]. In contrast, results of several studies support our findings. For example Renzi et al [10] demonstrated that tissue levels of SP were reduced in UC (n = 29 active and 39 inactive UC cases). Kimura et al [11] demonstrated that VIP and SP containing nerve fibers decreased in severe inflammatory lesions; increased in some hypervascular lesions, and remained unchanged in the uninvolved mucosa. Gross and Pothoulakis [12] demonstrated that SP and NK1R may also have protective roles in recovery from UC.

SP is a well-known pro-inflammatory neuropeptide [5–9]. In addition to its proinflammatory actions, SP and NK1R may play a protective role in mucosal regeneration and healing in the recovery phase of colitis. For example, Pavlovic et al [28] demonstrated that SP released during repeated stress exposure suppresses inflammation by increasing levels of IL-2 and T regulatory cells in a NK1R-dependent manner. Mice genetically deficient in NK1R was shown to have worse histologic and clinical signs of colitis and increased mortality in different colitis models [29]. These responses were associated with an SP-dependent epidermal growth factor receptor transactivation leading to proliferation in colonic fibroblasts and epithelial cells [30].

We demonstrated a simultaneous loss of NEP and SP at the same time in a pro-inflammatory condition. A possible explanation for the pro-inflammatory condition in low levels of SP may relate to bioactive fragmentation. It has been demonstrated that both N- and C-terminal fragments of SP have biological activity. The C-terminal portion of SP, SP (6–11), is known to interact with the NK1R as an agonist whereas the N-terminal SP fragment, SP (1–7) has been shown to attenuate or modulate several other effects of its parent peptide; acting as an antinociceptive, anti-inflammatory and anxiolytic molecule via an unknown receptor [31–37]. Given the facts that NEP-induced SP (1–7) inhibits inflammation and reduces pain, and SP (6–11), through NK1R activation, has healing properties in chronic inflammation, the decrease in SP (1–7) and SP (6–11) due to loss of NEP have a role in inflammation and increased pain in patients with UC.

Reduction of SP levels in UC could reflect degeneration of nerve endings containing SP and VIP as a consequence of a subclinical inflammation. Decreased NEP expression may reflect epithelial damage caused by local inflammation. Increased turnover of the peptides as the causative factor is less likely given the fact that the main enzyme responsible for hydrolysis is markedly decreased in UC. Further studies are required to determine causes resulting in the decreased peptide levels observed.

Different results in SP levels in patients with UC are possibly caused by different methodological approaches. A few studies which employed radioimmunoassay demonstrated increased SP levels in UC [5,6]. Other studies employed immunohistochemistry to show increased SP-containing nerve fibers [8,9]; whereas, another radioimmunoassay study showed decreased SP levels in colonic mucosa of patients with UC [10]. Differences in sample size might mask the limited changes in SP levels as well; since the decrease observed here was around 25%. Furthermore, different areas examined (e.g. active site vs inactive site, different levels of colonic mucosa) and different patient subpopulations (e.g. distal colitis vs pancolitis) may also create differences which was actually shown in the study by Kimura et al [11].

VIP, another neuropeptide hydrolyzed by NEP, is located in all layers of the colon; however it is primarily localized to neurons. VIP acts predominantly as an anti-inflammatory molecule affecting both innate and adaptive immunity, via its receptor called VPAC1 [38]. Treatment of experimental colitis with VIP was shown to effectively reduce severity of 2,4,6-trinitrobenzene sulfonic acid induced colitis in mice [39]. In addition, Vu et al demonstrated both VIP knock-out mice and VIP antagonist treated wild-type mice had remarkably reduced signs of dextran sodium sulfate induced colitis, further demonstrating that VIP has an anti-inflammatory role [40]. We here observed decreases in both neuronal and non-neuronal VIP levels in patients with UC. Our findings are in accordance with previous studies, which demonstrated that the loss of VIP is associated with colonic mucosal inflammation in patients with UC [6,10,14–17]. A concomitant loss of NEP may further reduce anti-inflammatory activity of VIP, since bioactive fragments of VIP have a higher affinity for VPAC1 as well [28].

In conclusion we here demonstrated concomitant decreases in SP and VIP with profound loss of NEP expression in colon samples from patients with UC. Our findings, in addition to previously published data, suggest an association between decreases in NEP and neuropeptide levels with the pro-inflammatory condition of colonic mucosa in patients with UC. Neuropeptide administration together with a recombinant NEP may hold therapeutic value which needs further study.

## Supporting information

S1 Fig Changes in CGRP levels (mean ± SEM). Levels were similar between healthy controls, healthy tissue samples of distal colitis and tissues with colitis.
(TIF)

S1 Dataset Main study data included as a.sav file. (SAV)

S2 Dataset Main study data included as an.xlsx file. (XLSX)
