# Supplementary figures and images for: Profound loss of neprilysin accompanied by decreased levels of neuropeptides and increased CRP in ulcerative colitis
Source: PLoS One. 2017 Dec 12;12(12):e0189526. doi: 10.1371/journal.pone.0189526 (PMC5726735; doi:10.1371/journal.pone.0189526)

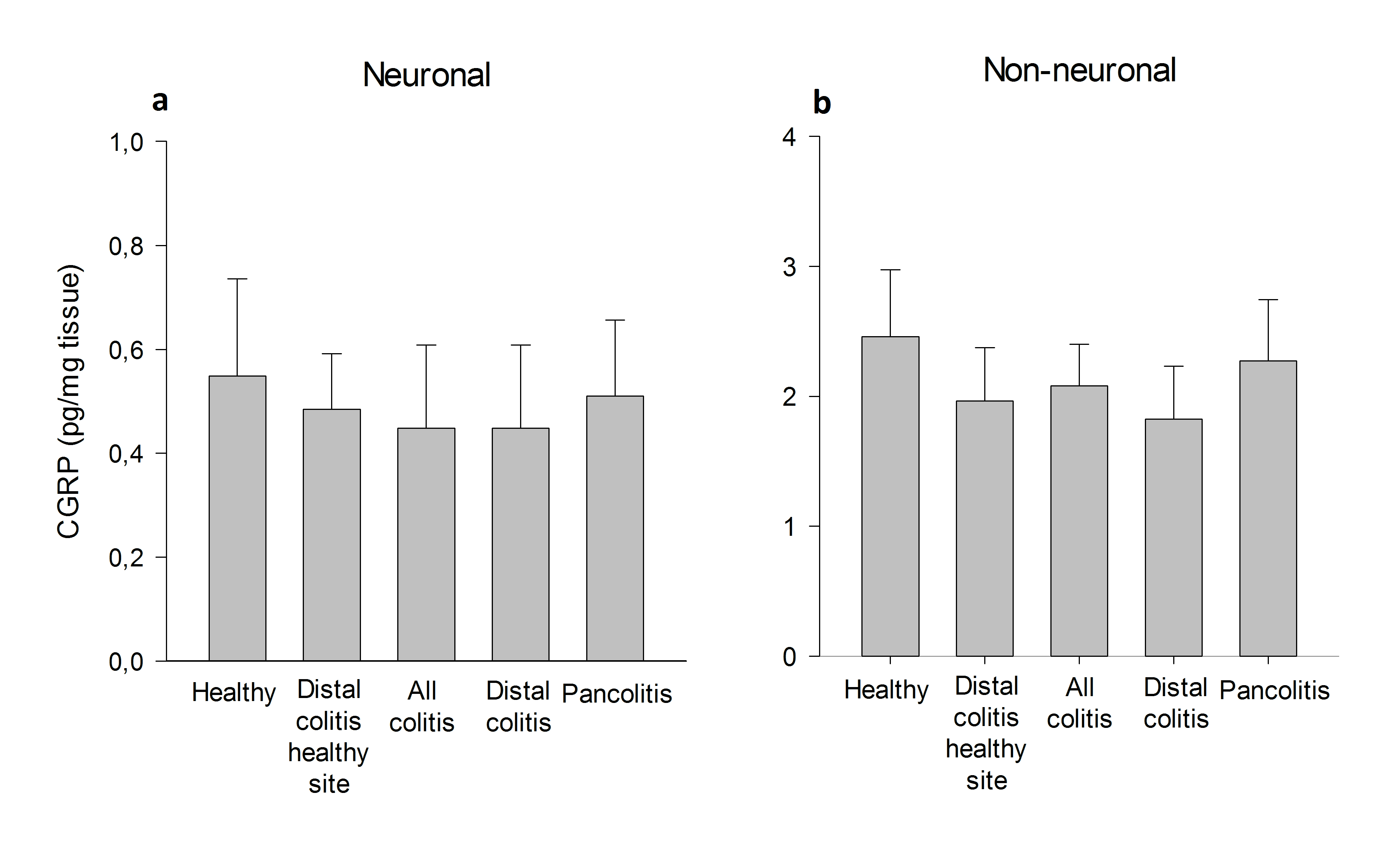

Supplement: S1 Fig — Levels were similar between healthy controls, healthy tissue samples of distal colitis and tissues with colitis. (TIF) [file pone.0189526.s001.tif]
